# Mega sporting events: A poisoned chalice or a new dawn for low- and middle-income countries?

**Published:** 2011-06

**Authors:** Mark Tomlinson

**Affiliations:** Department of Psychology, Stellenbosch University, South Africa

The increasing number of mega sporting events, such as Olympic Games, the World Cup or Commonwealth Games, awarded to low- and middle-income countries is, at first sight, a significant move in the direction of fairness and equity. In 2010, South Africa hosted the football World Cup and India hosted the Commonwealth Games, while Brazil will be hosting the football World Cup in 2014 and the Olympic Games in 2016. In South Africa, during the bidding process and in the lead up to the hosting of the World Cup, there was considerable commentary on the merits and otherwise of South Africa hosting the event, including benefits for the host country in particular and the continent in general. In the case of India, the Commonwealth Games was specifically marketed as an event that would improve ‘national prestige’ (1). In this brief viewpoint, using South Africa as a case study, I will outline a number of relevant health and economic issues associated with mega sporting events, and suggest that there are no tangible benefits to hosting these events, and that any intangible benefits (such as improving national prestige) are tenuous at best.

One of the central rationales for South Africa bidding to host the football World Cup, which was being voiced to the nation in many different shapes and forms, was the expected poverty relief that would be provided by the event, in the form of employment creation, infrastructure development and tourism and marketing. From the initial euphoria in 2004 when South Africa was awarded the rights to host the mega event, projections of tourist numbers and budget surpluses were significantly tempered in the run up to the event. The selling point for the 2010 World Cup in South Africa centered on government spending on infrastructure development and the expected tourist windfall. Organizers however, had to revise tourist estimates down from an initial 750 000 to between 200 000 and 250 000 (2). Tourism windfalls for mega events are often overstated – during the 2006 World Cup in Germany, despite large numbers of visitors (in line with expectations), hotel occupancy rates during the World Cup actually dropped (3).

It has been shown that while World Cup football is ‘extraordinarily’ profitable for international football association, FIFA, the economic projections for host countries usually overestimated the benefit and underestimated the cost (4), with some commentators arguing that in other mega sports events, such as the 2012 London Olympics, there is in fact a deliberate misrepresentation of costs and benefits (5). In terms of the misrepresentation of costs the 2010 World Cup in South Africa was an extreme example. In 2003, the estimate for the construction of stadiums was projected to be just over 1 billion rand (US$ 130 million, € 92 million), which by 2006 had ballooned to 8.5 billion rand (just over US$ 1 billion, € 0.7 billion) with the final costs likely to far exceed this figure (6). To put the figures in perspective, the cost of the Cape Town stadium was 3 times the entire housing budget for a single South African province in 2010. In the case of India, the initial budget of the Commonwealth Games was US$ 412 million (€ 291 million), with the eventual spend calculated to be in the region of US$ 15 billion (€ 11 billion) (7), while it has been reported that the residents of New Delhi will be paying for the Games in the form of increased prices of land, basic commodities and petrol for the next 25 to 30 years (8). Matheson and Baade (9) have argued that mega events are an even worse investment for low- and middle-income countries than for rich countries, and that net gains are invariably overestimated.

**Figure:**
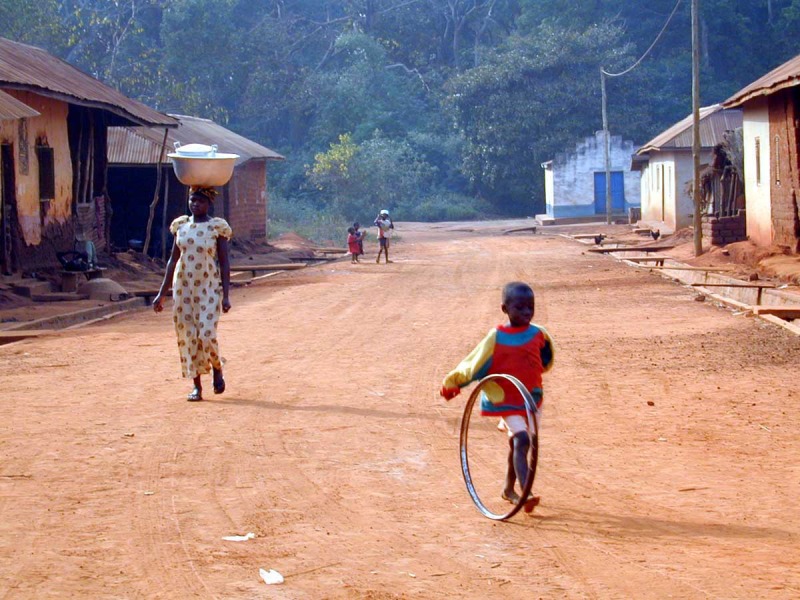
Photo: www.bigphoto.com

Collin and Mackenzie (10) have argued that there is a significant tension between international sport and health promotion when the image projected by FIFA of providing a legacy for health and contributing to societal development is in fact bankrolled by junk food producers and alcohol companies. While FIFA would never countenance the sponsorship of the World Cup by a cigarette company (in 2002, FIFA received an award from the WHO for their tobacco free policy), (11) they appear to have no ethical, economic or health objection to the event being sponsored by a beer company (Budweiser), junk food producers (Coke and McDonalds) or by a company that actively markets debt (Visa). This particular triangle of sponsors is an inappropriate one for countries such as South Africa and India, which are experiencing a health transition with simultaneous epidemics of infectious diseases, non-communicable diseases, as well as high levels of morbidity and mortality associated with alcohol related violence and motor vehicle accidents (12,13). South Africa has the highest rate of fetal alcohol disease in the world, with 7% of the country’s mortality rate attributable to alcohol abuse (14). In addition, South Africa has particularly high levels of obesity, with 56% of women being overweight or obese (15). Finally, South Africa’s debt levels are notably high (as a consequence of easily accessible credit) with consequent interest levels of 10%.

It is likely that the financial benefits for South Africa or India of hosting mega events will be negligible. It is also probable that hosting the event will not provide poverty relief as articulated in the rationale for hosting the World Cup, but rather it is likely that it will increase inequality both regionally and within cities (4). One of South Africa’s major cities (Port Elizabeth) has already begun a process of cutting back on services in order to try and service the debt incurred building a single World Cup stadium. The public relations nightmare that preceded India’s hosting of the Commonwealth Games, such as construction delays, bridges collapsing, poor facilities and corruption allegations (7), has surely undermined any benefit of the games for ‘national prestige’.

Having said this, South Africa has been described as a miracle nation (16), with the transition from apartheid to a democratic nation being a largely peaceful one. In 1995, South Africa won the rugby World Cup with the then President Nelson Mandela sporting a rugby jersey with the captain’s number on it. This moment was seen as a pivotal one in the process of South African nation building and beginning a process of unifying all South Africans. It has been argued that the true benefits of hosting the 2010 World Cup for South Africa were to celebrate African culture and to decrease Afro-pessimism (4). Such intangibles may be important and undoubtedly need to be considered. However, in the case of South Africa (largest number of HIV-positive people in the world) and India (1.8 million deaths of children under 5 each year and 52 million stunted children) (17), questions about the effective and (as importantly) the moral imperative of spending billions of dollar to host a sporting event must be asked. This, together with the questionable association, from a health perspective, with multinationals selling alcohol and debt, tarnishes further the notion of any intangible benefits of mega events to low- and middle-income countries.
